# Latin American Plants against Microorganisms

**DOI:** 10.3390/plants12233997

**Published:** 2023-11-28

**Authors:** Sofía Isabel Cuevas-Cianca, Cristian Romero-Castillo, José Luis Gálvez-Romero, Eugenio Sánchez-Arreola, Zaida Nelly Juárez, Luis Ricardo Hernández

**Affiliations:** 1 Department of Chemical Biological Sciences, Universidad de las Américas Puebla, Ex Hacienda Sta. Catarina Mártir S/N, San Andrés Cholula 72810, Mexico; sofia.cuevasca@udlap.mx (S.I.C.-C.); eugenio.sanchez@udlap.mx (E.S.-A.); 2 Biotechnology Faculty, Deanship of Biological Sciences, Universidad Popular Autónoma del Estado de Puebla, 21 Sur 1103 Barrio Santiago, Puebla 72410, Mexico; cristian.romero@upaep.edu.mx; 3 Chemistry Area, Deanship of Biological Sciences, Universidad Popular Autónoma del Estado de Puebla, 21 Sur 1103 Barrio Santiago, Puebla 72410, Mexico; 4 Department of Research ISSSTE Puebla Hospital Regional, Boulevard 14 Sur 4336, Colonia Jardines de San Manuel, Puebla 72570, Mexico; joseluis.galvez@upaep.mx

**Keywords:** Latin American plants, microorganisms, bacteria, fungi, virus

## Abstract

The constant emergence of severe health threats, such as antibacterial resistance or highly transmissible viruses, necessitates the investigation of novel therapeutic approaches for discovering and developing new antimicrobials, which will be critical in combating resistance and ensuring available options. Due to the richness and structural variety of natural compounds, techniques centered on obtaining novel active principles from natural sources have yielded promising results. This review describes natural products and extracts from Latin America with antimicrobial activity against multidrug-resistant strains, as well as classes and subclasses of plant secondary metabolites with antimicrobial activity and the structures of promising compounds for combating drug-resistant pathogenic microbes. The main mechanisms of action of the plant antimicrobial compounds found in medicinal plants are discussed, and extracts of plants with activity against pathogenic fungi and antiviral properties and their possible mechanisms of action are also summarized. For example, the secondary metabolites obtained from *Isatis indigotica* that show activity against SARS-CoV are aloe-emodin, β-sitosterol, hesperetin, indigo, and sinigrin. The structures of the plant antimicrobial compounds found in medicinal plants from Latin America are discussed. Most relevant studies, reviewed in the present work, have focused on evaluating different types of extracts with several classes and subclasses of secondary metabolites with antimicrobial activity. More studies on structure–activity relationships are needed.

## 1. Introduction

Infectious diseases are a significant source of public health issues. Despite breakthroughs in creating and manufacturing antivirals and antibiotics, bacteria, viruses, and other microorganisms continue to kill millions of people each year.

Antimicrobial resistance is a severe and developing clinical issue that has reduced the therapeutic effectiveness of conventional antibiotics and narrowed the treatment choices for bacterial infections. Antibiotic-resistant bacteria are generally difficult to treat due to reduced membrane penetration, efflux pump overexpression, target site shifting, inactive subpopulations, biofilm growth, and enzymatic destruction. Resistant bacteria are strains resistant to several medicines, resulting in increased infections [1].

Many bacteria may infect and live in their hosts for extended periods. This might be related to host immunosuppression, pathogen immune evasion, and/or inadequate drug clearance. Bacteria that are resistant or tolerant to antibiotics can survive treatment. Persistent bacteria are a transiently antibiotic-tolerant subset of bacterial cells that grow slowly or cease developing but can resume proliferation after exposure to fatal stress. Persistent cell production creates phenotypic variation within a bacterial population, significantly enhancing the odds of effectively responding to environmental change. The existence of resistant cells can lead to the emergence and recurrence of chronic bacterial infections and an increased risk of antibiotic resistance [2].

Emerging viral infections, on the other hand, continue to be a severe concern for worldwide public health. In 1997, it was revealed that a highly virulent avian influenza A (H5N1) virus may be transferred directly from poultry to people, in contrast to previously known human-to-human and livestock-to-human modes of transmission, raising severe fears about a probable influenza pandemic. Several additional avian influenza A virus subtypes (H7N9, H9N2, and H7N3) have also been linked to human sickness, increasing concerns that all influenza A virus subtypes circulating in domestic poultry and cattle in the wild might transmit to people and cause pandemics.

The most recent viral pneumonia epidemic, which began in mid-December 2019 (COVID-19) in Wuhan, China, and has spread swiftly throughout the world, is a stark reminder of our vulnerability to new viral illnesses. Tens of thousands of people are currently infected with SARS-CoV-2 [3].

In the case of fungus, it is believed that roughly 5 million species are extensively ubiquitous in the environment, of which approximately 300 can cause infections in people. However, only 20–25 are commonly seen in the clinic and are the cause of sick patients. Patients with HIV, organ transplant recipients, or those undergoing chemotherapy are examples of such people. The most frequent fungal diseases are *Candida* spp., *Cryptococcus* spp., *Aspergillus* spp., and *Pneumocystis* spp., which cause around 2 million illnesses and 1 million deaths yearly [4].

Because of the above, it is critical to enhance ways of treating infection-related disorders, preventing their spread, and filling the medicine shortage in order to alleviate this public health crisis. In an era of falling antimicrobial efficacy and the fast growth of antibacterial resistance, it is critical to develop novel therapies and tactics based on discovering new active components.

The exploration of active chemicals of natural origin is such potential methodology. Natural goods have served as a source of and the inspiration for many of the pharmaceuticals available today. Although numbers vary depending on the definition of what is deemed a medicine produced from a natural substance, it is reasonable to conclude that, today, natural products are the source of 25% to 50% of the pharmaceuticals on the market. The proportion is much more significant in the case of anti-cancer and anti-infective agents, with over two-thirds of such drugs originating from natural sources. Several recent reviews emphasize the importance of natural products in drug discovery. Many medicines in clinical use are derived from natural products that originated from microbial species, particularly in anti-infectives. However, drugs derived from plants have also made significant contributions. Humanity would undoubtedly be immeasurably poorer without plant-derived natural medicines such as morphine, vinblastine, vincristine, and quinine [5].

We provide a critical review of current research on natural product antibacterial activity and the discovery and classification of secondary metabolites of plants with antimicrobial activity, each with a distinct mechanism of action. The mechanisms of action of natural antifungal agents are also discussed, as are the potential antiviral mechanisms of biocompounds, which include viral replication inhibition through polymerases, proteases, integrases, fusion molecules, and cell membrane adhesion.

The Latin American plants presented in this review were selected from papers published in the last 20 years using databases such as SciFinder^®^, ScienceDirect^®^, Scopus^®^, PubMed^®^, PLOS, NATURE, and Google Scholar^®^. For the article search, the keywords “antimicrobial resistance”, “antibiotic resistance intrinsic”, “antibiotic resistance adaptive”, “antibiotic resistance acquired”, “antibiotic resistance mechanisms”, “antimicrobial activity of medicinal plants multidrug resistant bacteria”, “plant extract antimicrobial activity”, “plant extract multidrug resistant strains”, “plant extract antibiotic resistance”, “pathogenic fungi AND bioactive compounds”, “plant extracts AND pathogenic fungi”, “secondary metabolites AND fungal infections”, “drug resistance AND fungi”, “secondary metabolites against fungal infections” and “pathogenic fungi AND drug resistance” were used. The search in each database returned the following results: SciFinder^®^ (112 articles), ScienceDirect^®^ (556 articles), Scopus^®^ (1157 articles), PubMed^®^ (2365 articles), PLOS (552 articles), NATURE (409 articles), and Google Scholar^®^ (6354 articles). After a preliminary filter to collect only Latin American plants, 3827 articles were collected; of these, articles discussing non-specific antimicrobial (antibiotic, antifungal, and antiviral) activity were discarded. Only original papers and those published from 2003 to 2023 were considered for data collection.

## 2. Plant Antimicrobials

### 2.1. Antimicrobial Resistance

As COVID-19 rages, the antimicrobial resistance (AMR) epidemic continues in the background. AMR causes recurrent microbe (viruses, bacteria, and fungi) infections that lengthen hospital stays and result in preventable deaths. It is estimated that 4.95 million people died due to AMR in 2019 and that by 2050, there will be 10 million annual deaths due to antimicrobial resistance. Two factors primarily cause antimicrobial resistance. The first is the overuse of antimicrobials, which exposes microbes to them regularly, increasing their chances of developing resistance. The second issue is that few new antimicrobial drugs are being developed to replace ineffective ones due to rising drug resistance [6,7].

Compared to non-resistant forms, resistant bacteria are two times more likely to develop into a serious health problem and are three times more likely to lead to death [8,9]. Resistance to first-line antibiotics, such as fluoroquinolones and lactam antibiotics, is responsible for more than two-thirds of AMR-related deaths (carbapenems, cephalosporins, and penicillins). People with low incomes are disproportionately affected by AMR because they have limited access to more expensive second-line antibiotics that may be effective when first-line drugs fail. Physicians should avoid inappropriate antibiotic therapy when, for example, the illness has a viral origin [6,10,11].

**Figure 1:**
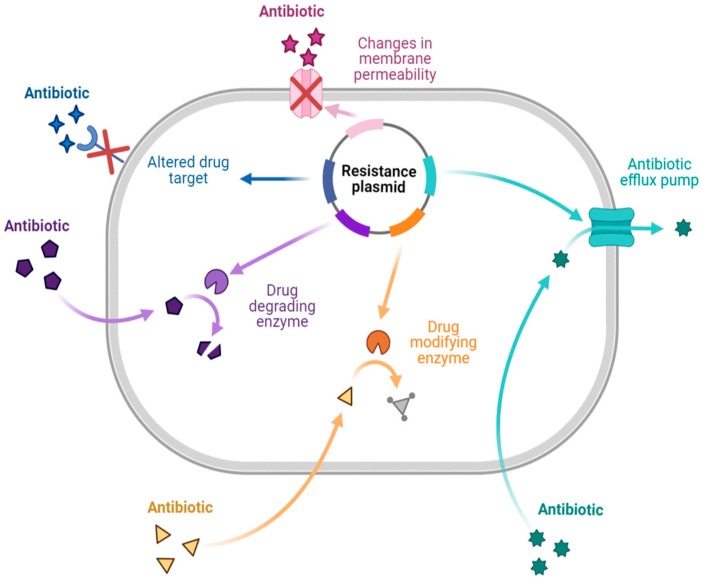
Antibiotic resistance mechanisms [7,12,13,14]. Created with BioRender.com.

There are different mechanisms of resistance to antibiotics (Figure 1). Bacteria produce enzymes that can destroy or alter the structure of the drug, causing the drug to lose its activity during enzymatic inactivation. Drug-inactivating enzymes are classified into three types: hydrolase (primarily lactamase), passivating enzymes (aminoglycoside-inactivating enzyme, chloramphenicol acetyltransferase, and erythromycin esterase), and modifying enzymes (aminoglycoside-modifying enzyme). Similarly, changing the target to which the drug is directed ensures that the antibiotic binds appropriately to the bacteria. This mechanism is primarily seen in Gram-positive bacteria with drug resistance and polymyxin resistance. Changes in outer membrane permeability that result in channel alteration or decreased expression make the bacteria less sensitive. In the drug efflux pump, when the drug is removed from the bacterial cytoplasm, the concentration is much lower than is required for it to exhibit activity, resulting in drug resistance. This process requires energy and works with various antibiotics [7,12,13,14].

### 2.2. Natural Products and Plant Extracts with Antimicrobial Activity against MDR Strains

Multidrug resistance (MDR) is a major cause of human suffering because it undermines doctor–patient trust, resulting in massive economic losses. In this world of microbe–man cohabitation, the survival of the human species will be compromised in the absence of health-giving microbes, and there will be no way to avoid the emergence of MDR superbugs. Throughout history, the isolation and identification of biologically active compounds and molecules from nature have resulted in the discovery of new therapeutics, advancing the health and pharmaceutical industries. Phytochemicals are used in the research and development of the pharmaceutical industry as a source of new molecules, leading to the development of novel drugs [15,16].

**Figure 2:**
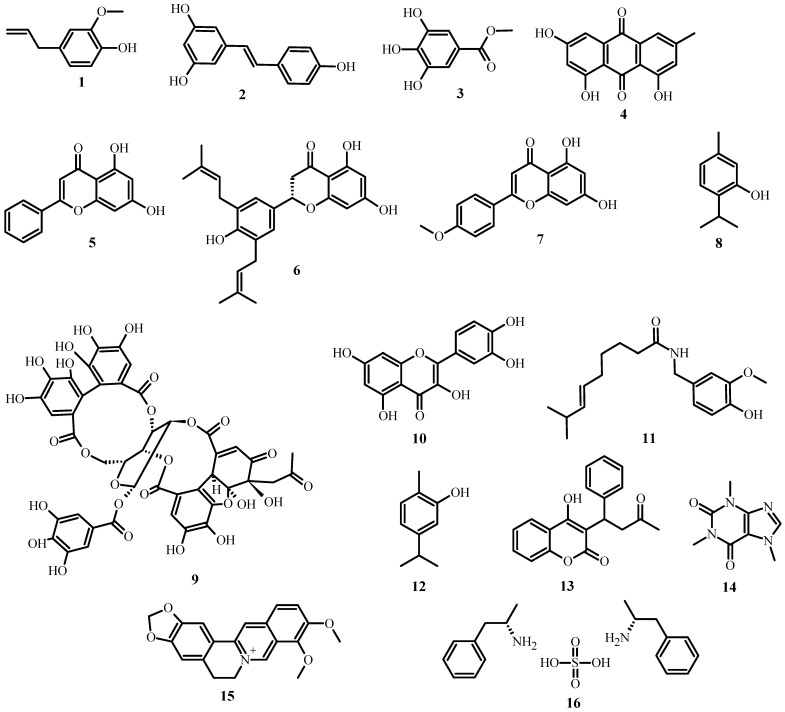
Structures of plant antimicrobial compounds found in medicinal plants from Latin America, from Table 1.

As shown in Table 1, several classes and subclasses of secondary metabolites (Figure 2) have been isolated from plants with antimicrobial activity, each with a different mechanism of action. This table shows that, depending on the compound class, they share the same kind of mechanism of action.

Regarding essential oils, the essential oil of rosemary (*Rosmarinus officinalis*) was found to have antibacterial activity against three types of MDR acne-causing bacteria: *Staphylococcus aureus*, *Staphylococcus epidermidis*, and *Cutibacterium acnes* [17]. Similarly, volatile oils extracted from cinnamon (*Cinnamomum verum)* and tree basil (*Ocimum gratissimum*) had potent bactericidal activity against MDR *A. baumannii* bacteria [18].

*Terminalia bellirica* fruits were studied, and it was discovered that the aqueous and methanol extracts had antibacterial activity against all strains of MRSA (Methicillin-resistant *Staphylococcus aureus*), MDR *Acinetobacter* spp., and MDR *P. aeruginosa* [19].

The aqueous, hexane, and ethanol extracts of *Punica granatum* peel demonstrated antibacterial activity against MDR pathogens such as *P. aeruginosa* and *A. baumannii*. Valoneic acid dilactone (aqueous fractions), Hexoside (ethanol fractions), and Coumaric acid (hexane fractions) were discovered to be bioactive compounds [18]. Ethanolic extracts of *Azadirachta indica*, *Allium sativum*, and *Syzygium cumini* were found to have anti-MDR-*Candida* spp. activity. According to a phytochemical analysis of ethanolic plant extracts, all the plants studied contained alkaloids, flavonoids, glycosides, phenols, tannins, and saponins [20].

ESKAPE (*Enterococcus faecium*, *Staphylococcus aureus*, *Klebsiella pneumoniae*, *Acinetobacter baumannii*, *Pseudomonas aeruginosa*, and *Enterobacter species*) MDR pathogens were tested using various extracts. Three ethanolic extracts from *Adiantum capillus-veneris*, *Artemisia absinthium*, and *Martynia annua* were found to inhibit the growth of MDR strains of ESKAPE pathogens [21].

**Figure 3:**
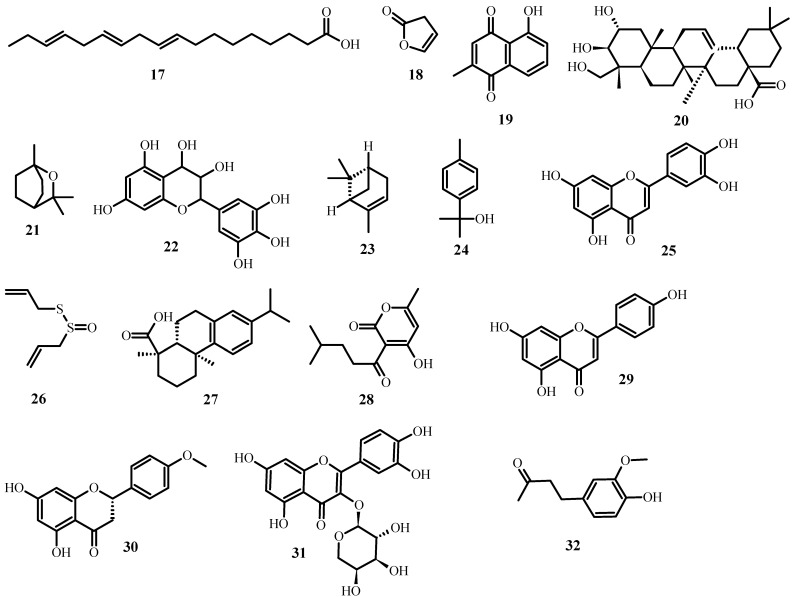
Structures of promising plant-derived compounds merit combating drug-resistant pathogenic microbes, from Table 2.

Aside from the plant extracts mentioned above, various plant compounds (Figure 3) with anti-MDR bacteria activity have already been identified. Table 2 lists these compounds, as well as the biological effects they have on specific strains.

Because of the severe problem of MDR properties in microbes, the discovery of alternative drugs from natural products should be one of the primary goals of current research. Understanding the nature of pathogenic microbes, recognizing biofilm formation and architectural scheme, and employing cross-disciplinary techniques are thus critical for discovering new potent and novel drugs.

**Table 1:** Antimicrobial mechanisms of plant compounds present in Latin American medicinal plants.

Class	Subclass	Examples	Source of the Compound	Mechanism	References
Phenolics	Simple phenols	Eugenol (**1**)	*Syzygium aromaticum*	Membrane disruption.	[22,23]
		Resveratrol (**2**)	*Vitis vinifera*	Binds reversibly to ATP synthase.	[22,24]
	Phenolic acids	Methyl gallate (**3**)	*Euphorbia hyssopifolia*	Inhibits DNA gyrase or ATPase.	[22,25]
	Quinones	Emodin (**4**)	*Rheum rhabarbarum*	Destroys the integrity of the cell wall and cell membrane.	[22,26]
	Flavonoids	Chrysin (**5**)	*Passiflora caerulea*	Binds to adhesins.	[27,28]
	Flavones	Abyssinone V (**6**)	*Erythrina abyssinica*	Complexes with the cell wall, inactivate enzymes and inhibit HIV reverse transcriptase.	[27]
		Acacetin (**7**)	*Robinia pseudoacacia*	-	[22]
	Flavonols	Quercetin (**10**)	*Brickellia cavanillesii*	Disrupts bacterial cell walls and cell membranes, disrupt nucleic acid synthesis, inhibit biofilm formation, and reduce expression of virulence factors.	[28,29]
	Tannins	Ellagitannin (**9**)	*Punica granatum*	Binds to proteins, bind to adhesins, enzyme inhibition, substrate deprivation, complex with the cell wall, membrane disruption, metal ion complexation.	[27]
	Coumarins	Warfarin (**13**)	*Melilotus officinalis*	Interacts with eukaryotic DNA (antiviral activity).	[27]
Terpenoids		Capsaicin (**11**)	*Capsicum annuum*	Membrane disruption.	[27]
		Carvacrol (**12**)	*Xylopia aromatica*	Membrane disruption.	[22,30]
		Thymol (**8**)		Induces the permeability and depolarization of the cytoplasmic membrane.	[22,31]
Alkaloids		Caffeine (**14**)	*Coffea arabica*	Inhibits biofilm development.	[22,32]
		Berberine (**15**)	*Argemone mexicana*	Damages bacterial cells by destroying cellular proteins.	[22,33]
Lectins and polypeptides		Fabatin (**16**)	*Vicia faba*	Blocks viral fusion or adsorption and forms disulfide bridges.	[27]

**Table 2:** Different compounds derived from plants with promising activity to combat drug-resistant pathogens (based on [34]).

Name of the Compound	Source of the Compound	Biological Effect on MDR Bacteria	References
9,12,15-Octadecatrienoic acid (**17**)	*Ocimum basilicum*	Used in contesting *E. coli*, *S. aureus*, *K. pneumonia*, *P. aeruginosa*, and *P. mirabilis.*	[34]
Furanone (**18**)	*Vanilla planifolia*	Interferes in the quorum sensing system of *P. aeruginosa.*	[35]
Plumbagin (**19**)	*Plumbago indica*	Has antibacterial properties by binding to the ATP cassette transporter.	[36,37]
Arjunolic acid (**20**)	*Cercidium microphyllum*	Inhibits *E. coli*, *B. subtilis*, and *S. sonnei.*	[38]
1,8-Cineole (**21**)	*Eucalyptus globulus*	Has antibacterial (methicillin-resistant *S. aureus*), antibiofilm, and anti-quorum sensing activities.	[39,40]
Leucoanthocyanidin (**22**)	*Umbellularia californica*	Has a cidal effect against *B. cereus* ATCC14579, *S. pyogens* ATCC10782, and *MRSA* ATCC-BAA-1683.	[41]
Quercetin (**10**)	*Citrus sinensis*	Inhibits the proton motive force (PMF) of *S. aureus* and inhibits *P. aeruginosa* (POA1), *E. coli* O157H7, and *V. harveyi* BB120.	[42]
Warfarin (**13**)	*Dipteryx odorata*	Inhibits *S. viridans*, *S. mutans* and *S. aureus.*	[16]
α-Pinene (**23**)	*Callistemon viminalis*	Suppresses the growth of *B. cereus*, *S. typhi*, *P. aeruginosa*, *B. subtilis*, *E. coli*, and *P. vulgaris.*	[43]
*p*-Cymen-8-ol (**24**)	*Senecio nutans*	Interferes with the membrane permeability of *V. cholerae.*	[44]
Luteolin (**25**)	*Guazuma ulmifolia*	Has a cidal effect against *M. tuberculosis*.	[45]
Allicin (**26**)	*Allium sativum*	Interferes with the metabolic systems of *H. pylori*, *S. epidermidis*, *B. cepacia*, *P. aeruginosa*, and *S. aureus.*	[46]
Thymol (**8**)	*Lippia sidoides*	Has activity against *L. monocytogen*, *S. typhimurium*, and *E. coli* O157:H7.	[46,47]
Dehydroabietic acid (**27**)	*Pinus elliottii*	Has a cidal effect against *E. faecalis*, *S. haemolyticus*, *S. capitis*, and MDR-*S. epidermidis.*	[48]
Pogostone (**28**)	*Pogostemon cablin*	Is effective against both gram-negative and gram-positive bacteria.	[49]
Apigenin (**29**)	*Mentha pulegium*	Interferes with the growth of *B. cereus*, *E. coli*, and *S. aureus.*	[50]
Isosakuranetin (**30**)	*Hyptis albida*	Inhibits *S. aureus* and *B. subitilis.*	[51]
Guaijaverin (**31**)	*Psidium guajava*	Significantly inhibits the adherence of *S. mutans.*	[52,53]
Zingerone (**32**)	*Zingiber officinale*	Inhibits biofilm formation and attenuation of motility properties in *P. aeruginosa.*	[54,55,56]

### 2.3. Pathogenic Fungi for Human

Fungi are eukaryotic organisms widely distributed across the planet, with more than 700,000 species classified [57]; however, it is estimated that there may be more than 1 million species in existence [58]. Despite these data, the number of fungi that can affect other species is minimal, with less than 0.1% being of medical importance to humans, and less than 50 species being identified as pathogenic fungi. In recent years, fungi adapted to modified ecosystems have significantly impacted human health, as they tend to infect plants and their metabolism, negatively affecting the food web [59,60].

Mycoses are usually superficial, cutaneous, systemic, or opportunistic. A worldwide risk factor is immunosuppression; however, the microbiome imbalance caused by antibiotics must be considered, as it can lead to an even more severe infection [61,62]. It is widely thought that most mycoses are opportunistic. It is extremely important to take into account that mycosis can be considered dangerous due to the entry of several fungi, with cosmopolitan genera such as *Candida*, *Cryptococcus*, and *Aspergillus* being prevalent [63,64,65], while creating invasive fungal infections (IFIs) that cause high mortality rates worldwide [60,66,67].

### 2.4. Mechanism of Action and Drug-Resistance of Pathogenic Fungi

Pathogenic fungi create complex signaling cascades that depend on the host and environment [68]. A 2017 review points out the importance of recognizing the pathways involved in fungal pathogenicity and identifying opportunity areas to create better antibiotics [69], even if knowing these factors would make it impossible to create efficient vaccines [70,71]. However, current antifungal drugs have different mechanisms of action (Table 3); the most common mechanisms are directed against the fungal cell wall or membrane, specifically against ergosterol or (1,3)-β-d-glucan biosynthesis, except for pyrimidines and orotomides that target crucial molecules in nucleic acid metabolism [72,73,74,75].

Just as bacteria generate drug resistance, so do fungi; this drug resistance can be described from a clinical point of view, referring to the worsening of an infection despite receiving adequate drug treatment. On the other hand, in the laboratory context, resistance is evaluated through a Minimum Inhibitory Concentration (MIC) assay to determine the growth of the pathogen at different concentrations of antibiotics [68,69,78]. It is necessary to point out the concept of drug tolerance, which is considered as the fungus persistence on the substrate; however, its growth is slow due to multifactorial causes [79,80].

### 2.5. Latin American Plants with Antifungal Effects

Fungi drug resistance has created a worldwide clinical challenge, and treatment alternatives have been considered, such as including two or more antifungals for one treatment; however, this does not make a significant difference [81]. This is why alternatives should be considered, such as using plant-derived compounds that can act via bypassing common metabolic pathways in fungal pathology. Table 4 summarizes the medicinal plant extracts with antifungal properties.

### 2.6. Medicinal Plant Antiviral Activity against Human-Infecting Viruses

In 2018, over 4400 virus species were classified into 122 families and 7535 [111] subfamilies. Human-infecting viruses include RNA viruses, DNA viruses, retroviruses, bare viruses, and virions, with RNA viruses being the most prevalent. Numerous medicinal plants contain compounds that inhibit the replication of viruses or enhance the immune system. Alkaloids, terpenes, flavonoids, numerous glucosides, and proteins have been recognized as phytochemicals; their metabolites include apigenin (**29**), kaempferol (**34**), and luteolin (**25**), in addition to the triterpenoids oleanolic acid (**35**) and ursolic acid (**36**) [112].

#### 2.6.1. Biological Mechanisms of Antiviral Activity

Plant biocompounds may function similarly to conventional antiviral medications by inhibiting viral replication polymerase, protease, integrase, fusion molecules, and cell membrane binding. For example, the exposure of non-enveloped norovirus to 0.5% of carvacrol (**12**) results in the degradation of its capsid [113]. Some polysaccharides may also deter viruses from attaching to cells, while thiophenes, terpenoids, and polyacetylenes can interact with the membrane of infected cells [114]. Lignans, phenolic compounds, terpenoids, flavonoids, alkaloids, and furocoumarins can all inhibit viral replication. Biocompounds of *Allium sativum* are among the most studied; they have antiviral activity against human, animal, and plant infections. Multiple of these metabolites can strengthen the immune system’s response to infections. This biocompound interacts in vivo with thiols such as glutathione and L-cysteine to produce S-allyl-mercapto-glutathione (SAMG) and S-allyl-mercapto-cysteine (SAMC), which can degrade viral protein. In addition, it contains lectins, flavonoids (kaempferol (**34**), quercetin (**10**), and myricetin (**37**)), polysaccharides (fructan), steroids, saponins, fatty acids (lauric (**38**) and linoleic acid (**39**)), diverse enzymes, vitamins (A, B1, and C), allixin (**40**), minerals (Ca, Cu, Fe, K, Mg, Zn, and Se), and amino acids [115].

**Figure 4:**
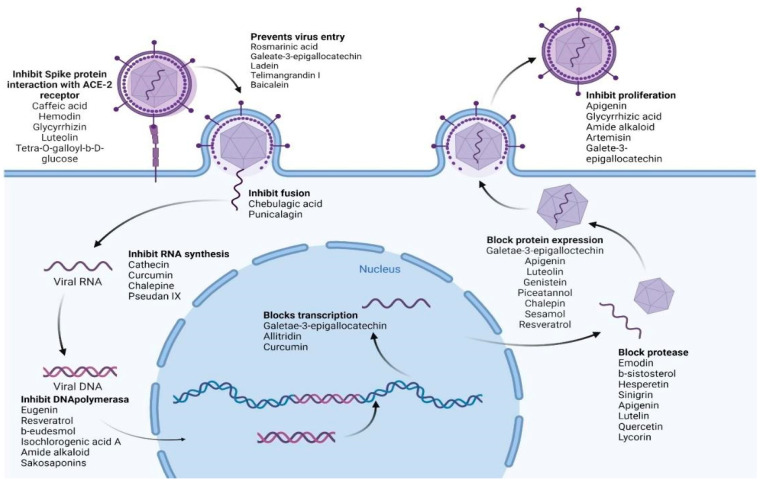
Various biocompounds’ potential antiviral mechanisms of action. Created with BioRender.com.

**Figure 5:**
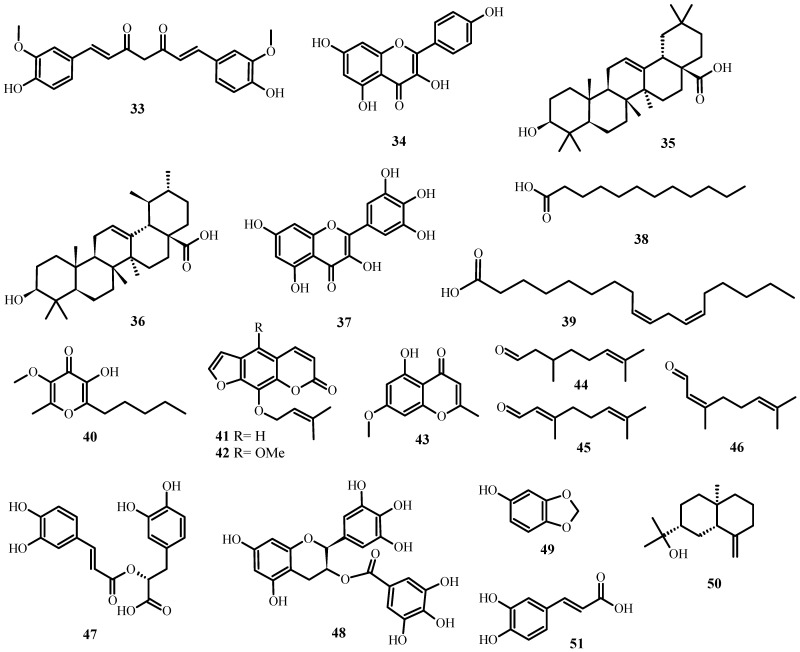
Antiviral biological compounds (Table 6).

**Figure 6:**
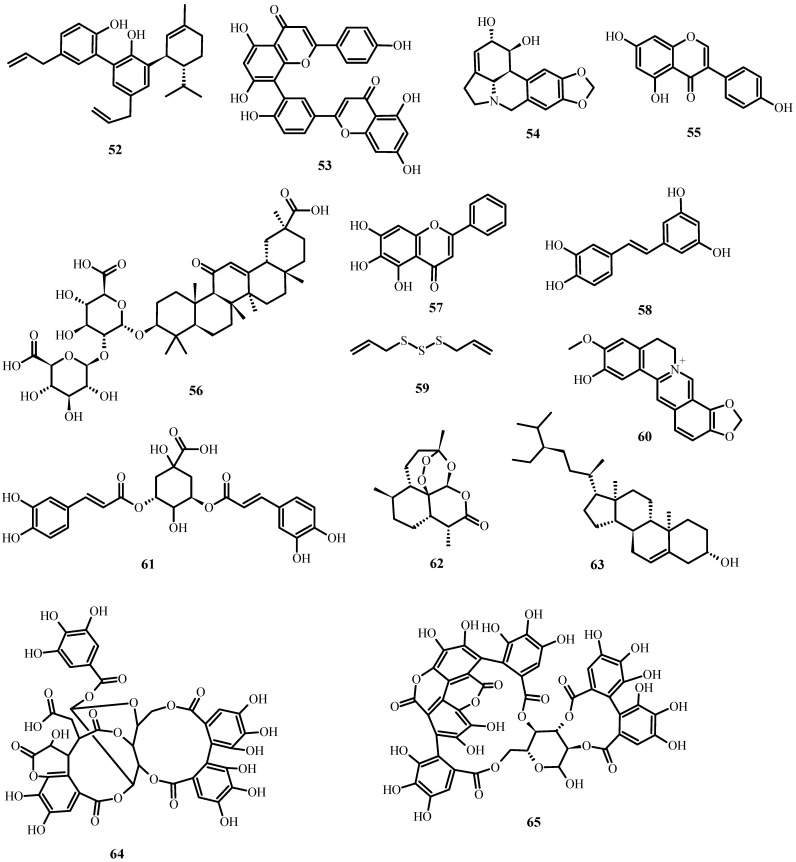
Antiviral biological compounds (Table 6).

**Figure 7:**
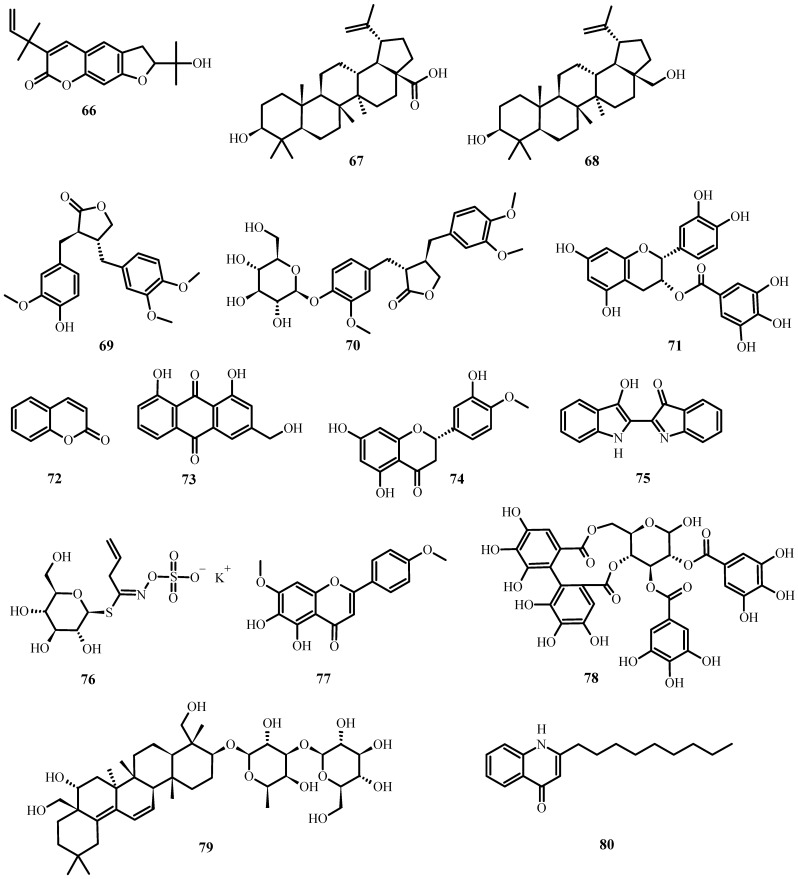
Antiviral biological compounds (Table 6).

Table 5 summarizes the medicinal plant extracts and their possible mechanisms of action (Figure 4), while Table 6 discusses the medicinal plant biocompounds with antiviral properties (Figure 5, Figure 6 and Figure 7).

#### 2.6.2. Antiviral-Active Extracts for Respiratory Infections

The leading cause of morbidity in humans is viral respiratory tract infections, with rhinovirus, influenza, respiratory syncytial virus (RSV), and human coronavirus having the most significant impact.

Several extracts of medicinal plants exhibit antiviral activity in vitro; the ethanolic, ethyl acetate, and hexane extracts of *Echinacea pallida* var. *angustifolia* root inhibit rhinovirus replication [116]. The *Echinacea purpurea* ethanolic extract inhibits the invasion of HcoV-299E (coronavirus) into cells [117]. The ethanolic extract of *Sambucus formosana* Nakai seeds inhibits the binding of HCoV-NL63 (coronavirus) [118]. Aqueous extracts of *Plantago asiatica* and *Clerodendrum trichotomun* inhibit RSV (respiratory syncytial virus) replication [119].

#### 2.6.3. Extracts and Biocompounds with Activity against Human Herpes Viruses

Several medicinal plant extracts have in vitro anti-herpes simplex activity: the hexane, dichloromethane, and methanolic extracts of *Clinacanthus mutans* and *C. siamensis* inhibit the formation of HS-1 and HS-2 viral plaques [120]. Caffeic acid and chlorogenic acid are inhibitors of HS replication [121]. *Polygonum minus* methanolic extract inhibits HS adhesion [122]. *Aloe vera* glycerol extract prevents HS-2 replication [123]. *Lysimachia mauritiana* ethanolic extract inhibits varicella-zoster virus replication [89].

Alkaloids, glycosides, taxol derivatives, terpenes, flavonoids, ellagitannin, catechin, phenolic acids, triterpenoids, monoterpenoids, and steroids have been identified as active against Herpes simplex types 1 and 2 [124].

Carvacrol (**12**), extracted from the essential oil of Mexican oregano (*Lippia graveolens*), demonstrates antiviral activity against RNA and DNA viruses (primarily herpes viruses) [125].

Coumarins imperatorin (**41**) and phellopterin (**42**), isolated from *Angelica archeangelica* L., exhibit antiviral activity against herpes simplex virus type 1 and, most likely, Coxsackievirus B3 [126].

Eugenin (**43**) is a biocompound extracted from *Geum japonicum* and *Syzygium aromaticum*. Eugenin (**43**) inhibits the DNA polymerase of the Herpes simplex virus, which appears to be its mechanism of action. Also, it inhibits Herpes simplex virus activity in both Vero cells and mice [127].

The monoterpene aldehydes citral a (**45**), citral b (**46**), and citronellal (**44**) are the biocompounds found to have anti-herpes virus activity in *Melissa officinalis* essential oil [128]. Moreover, rosmarinic acid (**47**) from the hydroalcoholic leaf extract of *M. officinalis* demonstrates anti-herpes simplex type 2 activity [129]. The potential mechanism of action is to prevent virus entry into cells [130].

#### 2.6.4. Activity against Epstein-Barr Virus

Epstein–Barr (EBV) is a herpes virus that affects 90 percent of the world’s population and is linked to numerous immunological and neoplastic diseases.

Epigallocatechin-3-gallate (**48**), a catechin derived from *Camellia sinensis*, inhibits the spontaneous lytic infection of infected cells and blocks their transcription and protein expression via the ERK1/2 (extracellular-regulated kinase 12) and PI3-K/Akt (phosphatidylinositol-3-kinase) pathways [131].

The compounds sesamol (**49**) and resveratrol (**2**), along with sesame and sunflower essential oils, inhibit the early antigen activation in vitro of the Epstein–Barr virus [132].

Konoshima et al. [133] found that monoterpenylmagnolol (**52**) and β-eudesmol (**50**), extracted from *Magnolia officinalis*, inhibit replication (EBV) in Raji cells.

Berberine (**15**) is an alkaloid derived from several medicinal plants (*Cortidis rhizome*, *Coptis chinensis*, and *Barnerini vulgaris*) that inhibits cell proliferation and induces apoptosis in Epstein–Barr virus-infected cells via the inhibition of p-STAT3 and the overexpression of EBNA1 [134].

Curcumin (**33**) is highly effective at reducing TPA-, butyrate-, and TGF-b-induced levels of BZLF1 mRNA and TPA-induced luciferase mRNA, indicating that it inhibits three main EBV pathways [135].

Apigenin (**29**) inhibits the expression of the EBV lytic proteins Zta, Rta, EAD, and DNase in B and epithelial cells. In addition, it decreases the number of EBV-reactivating cells detectable via immunofluorescence analysis. Additionally, apigenin (**29**) has been found to significantly reduce EBV virus production [136].

Glycyrrhizic acid (**56**) (18-GL or GL) possesses a wide range of antiviral activities, pharmacological effects, and sites of action. In vitro, GL (**56**) inhibits Epstein–Barr virus (EBV) infection by interfering with an early stage in the EBV replication cycle (possibly attachment or penetration) [137].

The flavonoid luteolin (**25**) inhibits EBV reactivation significantly. In EBV-positive epithelial and B cell lines, **25** inhibits the expression of EBV-lytic gene-encoded proteins. In addition, it decreases the number of EBV-reactivating cells detected via immunofluorescence and virion production. Moreover, **25** decreases the activities of the promoters of the immediate–early genes Zta (Zp) and Rta (Rp). It inhibits the activity of Sp1-luc, indicating that the disruption of Sp1 binding is involved in the mechanism of inhibition [138].

#### 2.6.5. Anti-Cytomegalovirus Activities

Human cytomegalovirus (hCMV) is a pervasive herpesvirus that causes a latent infection that persists throughout the host’s lifetime and can be reactivated when immunity is compromised.

Genistein (**55**) and baicalein (**57**) are antiviral flavonoids against HCMV. The primary mode of action of genistein’s antiviral activity against HCMV is to inhibit the function of immediate–early proteins. Baicalein’s antiviral activity against HCMV works primarily by inhibiting the kinase activity of EGFR to prevent viral entry [139].

Supplementation with piceatannol (**58**) inhibits the lytic changes caused by hCMV infection. In addition, piceatannol dose-dependently inhibits the expression of hCMV immediate–early (IE) and early (E) proteins and the replication of hCMV DNA [140].

Resveratrol (**2**) inhibits human cytomegalovirus DNA replication to undetectable levels during the second (late) phase of virus-induced phosphatidylinositol-3-kinase signaling and transcription factor activation [141].

Allitridin (**59**), a compound extracted from *A. sativum*, reduces the amount of viral DNA in cytomegalovirus-infected cells by inhibiting the transcription of the IE gene [142].

#### 2.6.6. Anti-HIV Activity of Extracts and Biocompounds

Among the extracts that inhibit in vitro HIV activity or replication is an aqueous extract of *Salvia miltiorrhiza* that inhibits HIV-1 integrase [143]. *Rhaphiolepsis indica* methanolic extract inhibits its replication [144]. *Acacia arabica*’s n-butanol fraction inhibits the activity of viral proteases and Tat [145]. The *Phyllanthus amarus* ethanolic and aqueous extracts inhibit its replication [146]. The *Olea europaea* aqueous extract inhibits cell–cell infection [147]. *Hyssopus officinalis* L. aqueous extract inhibits its replication [148]. The reverse transcriptase is inhibited by the methanolic extract of *Terminalia sericea* [149], the n-hexane fraction of *Phyllanthus emblica* and *Cassia occidentalis*, and the pine cone extract of *Pinus yunnanensis* [150]; floral extracts of *Calendula officinalis* inhibit HIV-1 reverse transcriptase activity [151]. *Cassine xylocarpa*’s lupane-type pentacyclic triterpenoid also possesses anti-HIV activity [152].

#### 2.6.7. Antiviral Activity of Extracts and Biocompounds against Hepatitis B and C Viruses

The secondary metabolites and extracts listed below have demonstrated in vitro activity against HBV (Hepatitis virus B): isochlorogenic acid A (**61**), obtained from *Laggera alata*, inhibits replication and decreases the stability of its core protein [153]. Amide alkaloids from *Piper longum* [154] and dehydrocheilanthifoline (**60**), isolated from *Corydalis saxifolia*, inhibit its replication [155]. Saikosaponins (*Bupleurum* species) inhibit the replication and expression of its surface antigen [156]; the ethanolic extract of *Polygonum cuspidatum* inhibits its surface antigen expression [157]; and curcumin (**33**) (*Curcuma longa*) decreases the expression of the PGC-1a coactivator, required for its transcription [158]. Glycyrrhizinic acid (**56**) (*Glycyrrhiza glabra*), artemisinin (**62**) (*Artemisia annua*), and LPRP-Et-97,543 compound (*Liriope platyphylla*) all inhibit its viral production [159]. On the other hand, epigallocatechin-3-gallate (**48**) (*Camellia sinensis*) inhibits its viral replication [160].

Concerning the hepatitis C virus, flavonolignans (*Silybum marianum*) possess antiviral and antioxidant properties [161], while curcumin (**33**) (*Curcuma longa*) inhibits its viral replication via the Akt-SREBP-1 pathway [162]. Epigallocatechin-3-gallate (**48**) [163] and ladanein (**77**) [164] inhibit viral entry; griffithsin inhibits viral cell–cell transmission [165], tellimagrandin I (**78**) (*Rosa rugosa*) inhibits viral invasion [159], chebulagic acid (**64**) and punicalagin (**65**) (*Terminalia chebula*) inhibit the viral particles necessary for their fusion and cell–cell transmission [166], saikosaponin B2 (**79**) (*Bupleurum kaoi*) prevents viral binding, and chalepine (**66**) and pseudan IX (**80**) (*Ruta angustifolia*) decrease viral protein synthesis and RNA replication [159,167].

Betulinic acid (**67**) and betulin (**68**), derived from *Betula alba* L., exhibit anti-hepatitis C virus activity. Shikov et al. (2011) [168] suggested that **68** can induce TNF-α expression and thereby enhance the Th1-type immune cell response in patients with chronic hepatitis C virus.

#### 2.6.8. Anti-Influenza Activity of Extracts and Biomolecules

Moradi (2019) [169] reported that ethanolic and polyphenolic extracts of *Punica granatum* inhibit influenza replication and virions. *Geranium sanguineum* polyphenolic, methanolic, and ethanolic extracts have antiviral properties [170]. Glycyrrhizin (**56**) from *Glycyrrhiza glabra* induces the apoptosis of H5N1-infected cells [171]; polyphenols from *Chenomeles sinensis* inhibit the binding of its hemagglutinins [172]; and the *Sambucus nigra* fruit inhibits viral entry and modulates cytokine release. It has been shown in other studies to inhibit hemagglutins and the replication of influenza viruses: A/Shangdong 9/93 (H3N2), A/Beijing 32/92 (H3N2), A/Texas 36/91 (HlNl), A/Singapore 6/86 (HlNl), type B/Panama 45/90, B/Yamagata 16/88, and B/Ann Arbor [173].

The *Phyllanthus embolica* aqueous extract inhibits hemagglutinin and viruses in infected cells [174]. Catechin derived from *Camellia sinensis* inhibits both RNA synthesis and neuraminidase activity [175].

Arctigenin (**69**) and arcitiin (**70**), extracted from the fruits of *Arctium lappa* L., exhibit potent anti-influenza A virus activity in vitro [176].

*Echinacea* extract is active against influenza A/B viruses (H3N2, H1N1, H5N1, H7N7, and S-OIV), Respiratory Syncytial Virus, and Herpes Simplex [177]. On the other hand, it also induces the production of IL-6 and IL-8 (CXCL8) and other cytokines with antiviral properties [178]. In a clinical trial, it was demonstrated to be as effective as oseltamivir in reducing influenza symptoms if administered at the onset of the disease [179].

On the other hand, the monoterpene aldehydes citral a (**45**) and citral b (**46**), from *Melissa officinalis*, exhibit synergistic activity with oseltamivir against the H9N2 influenza virus [180].

Wyde et al. [181] found that polyphenolic polymers derived from the Euphorbiaceae shrub are active in vitro against parainfluenza virus type 3, Respiratory Syncytial Virus, and influenza viruses.

#### 2.6.9. Extracts In Vitro Possess Anti-Papillomavirus Activity

Their growth is inhibited by polyphenon E (**71**) (poly E) and epigallocatechin gallate (**48**) from *Camellia sinensis* [182]. Artemisinin (**62**) (*Artemisia absintium*) inhibits the expression of HPV-39, induces apoptosis, and reduces the proliferation of infected cells in ME-180 cells [183]. Curcumin (**33**) (*Curcuma longa* L.) has been utilized to boost immunity against HPV. *Hamamelis virginiana* tannins inhibit HPV-16, *Ficus religiosa* aqueous extract induces the apoptosis of HPV-16 and 18 infected cervical cells, and the *Phyllanthus emblica* fruit inhibits HPV-16 and 18 carcinogenic gene expression. The chloroplast leaf extract of *Bryophyllum pinnatum* inhibits HPV-18 transcription in cervical cancer cells, whereas the soluble extract of *Pinellia pedatisecta* inhibits HPV-E6 expression in multiple cell lines [184].

#### 2.6.10. In Vitro Activity of Extracts against Dengue and Chikungunya Viruses

Coumarin (**72**) and the ether extract of *Alternanthera philoxeroides* [185], as well as aqueous and chloroform extracts of *Carioca papaya* [186], inhibit dengue virus. *Sambucus nigra* methanolic extract protects against dengue serotype 2 [187].

*Vernonia amygdalina* ethyl acetate extract reduces the Chikungunya viral burden [188]. Chikungunya helicases and proteases are inhibited by aqueous extracts of *Picrorhiza kurrooa*, *Ocimum tenuiflorum*, and *Terminalia chebula* [189].

#### 2.6.11. Antiviral Activity of In Vitro Extracted Compounds against SARS-CoV

The following organisms were evaluated for their anti-SARS-CoV-1 activity: *Lycoris radiate*, *Artemisia annua*, *Pyrrosia lingua*, *Lindera aggregata*, *Isatis indigotica* (inhibition of 3CL protease) [190], *Rheum officinale* Bail, *Polygonum multiforum* Thunb. (inhibit ACE2 protein interaction with spike protein) [191], *Gentiana scabana*, *Dioscorea batatas*, *Casssia tora*, *Taxillus chinensis*, *Cibotium barometz* (inhibit 3CL protease) [192], and ethanolic extracts of *Anthemis hyalina*, *Nigella sativa*, and *Citrus sinensis* (increase IL-8, modify TRPA, TRPM, and TRPV gene expression) [193]. Some purified secondary metabolites that show activity against SARS-CoV are as follows: aloe-emodin (**73**), β-sitosterol (**63**), hesperetin (**74**), indigo (**75**), and sinigrin (**76**) (obtained from *Isatis indigotica*) [194], amentoflavone (**53**), apigenin (**29**), luteolin (**25**), quercetin (**10**) (obtained from *Torreya nucifera*), which inhibit 3CL protease [195], and lycorine (**54**) (obtained from *Lycoris radiata*) [190].

**Figure 8:**
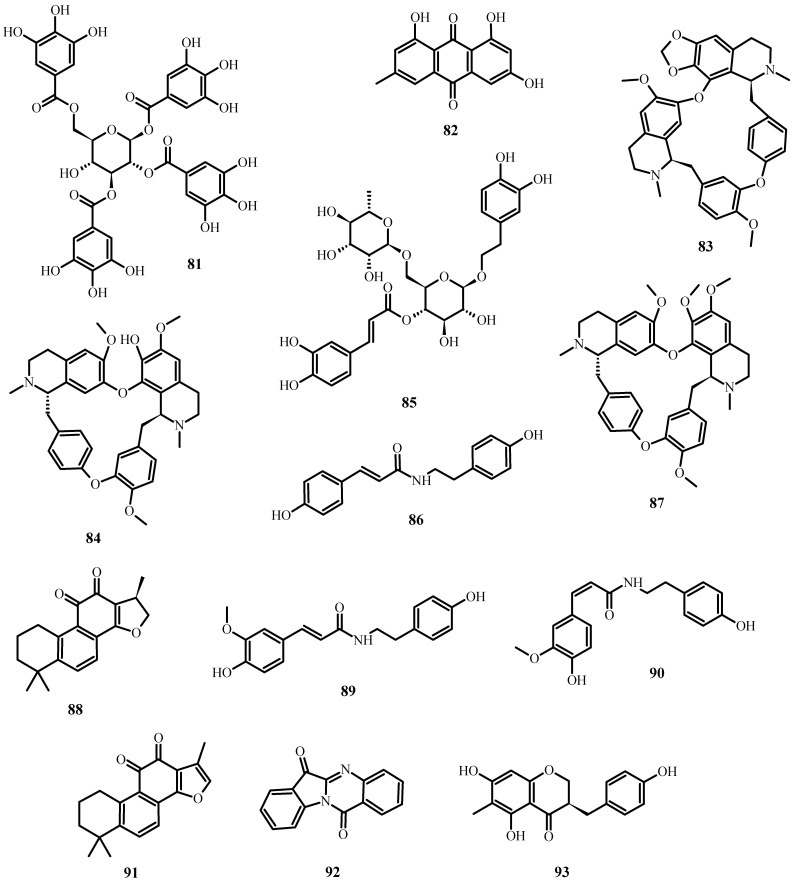
Natural products reported to have anti-SARS-CoV-2 activity.

In the case of SARS-CoV-2 (which causes COVID-19), the following secondary metabolites (Figure 8) may be advantageous [196]:

1. To inhibit the binding of the spike protein to the ACE-2 receptor: caffeic acid (**51**), emodin (**82**), glycyrrhizin (**56**), luteolin (**25**), and tetra-O-galloyl-β-D-glucose (**81**).
2. To prevent virus transcription: cepharanthin (**83**), fangquinoline (**84**), forystoside A (**85**), tetrandin (**87**), coumaroyltyramine (**86**), cryptoansionone (**88**), kaempferol (**34**), moupinamide (**89**), N-*cis*-feruloyltyramine (**90**), quercetin (**10**), tanshinone IIa (**91**), and tryptanthrine (**92**).
3. To inhibit viral translation: tryptanthrine (**92**).
4. To inhibit the cellular discharge of virions: emodin (**82**) and kaempferol (**34**).

**Table 5:** Antiviral extracts derived from plants.

Plant	Extract	Virus	Possible Antiviral Mechanism	References
*Echinacea pallida* var. *angustifolia*	Hexane	Rhinovirus	Impedes replication.	[116]
*Echinacea purpurea*	Ethanolic	Coronavirus HcoV-299E	Prevents the invasion of cells.	[117]
*Sambucus formosana* Nakai	Ethanolic	HCoV-NL63 (coronavirus)	Prevents bonding.	[118]
*Plantago asiatica*	Aqueous extract	Respiratory syncytial virus	Replication inhibition.	[119]
*Clerodendrum trichotomun*	Aqueous extract	Respiratory syncytial virus	Replication inhibition.	[119]
*Clinacanthus mutans* *Clinacanthus siamensis*	Hexane, dichloromethane, and methanolic	Herpes simplex-1 and 2	Inhibit viral plaques.	[120]
*Polygonum minus*	Methanolic	Herpes simplex-1 and 2	Inhibits adhesion.	[122]
*Aloe vera*	Glycerol	Herpes simplex 2	Impedes replication.	[123]
*Lysimachia mauritania*	Ethanolic extract	Varicella-zoster	Impedes replication.	[89]
*Sesamum indicum* *Helianthus annuus*	Sesame essential oil and Sunflower essential oil	Epstein-Barr Virus	Inhibit precocious antigen activation.	[132]
*Salvia miltiorrhiza*	Aqueous extract	HIV-1	Interferes with integrase activation.	[143]
*Rhaphiolepsis indica*	Methanolic extract	HIV-1	Impedes replication.	[144]
*Acacia arabica*	N-butanol fraction	HIV-1	Inhibits viral proteases and Tat activity.	[145]
*Phyllanthus amarus* Schum.	Ethanolic and aqueous extract	HIV-1	Impedes replication.	[146]
*Olea europaea*	Aqueous extract	HIV-1	Prevents infections between cells.	[147]
*Hyssopus officinalis* L.	Aqueous extract	HIV-1	Inhibits replication.	[148]
*Polygonum cuspidatum*	Ethanolic extract	Hepatitis virus B	Inhibits surface antigen expression.	[157]
*Punica granatum*	Ethanolic and polyphenolic extracts	Influenza virus	Inhibits influenza replication and virions.	[169]
*Geranium sanguineum*	Polyphenolic, methanolic, and ethanolic	Influenza virus	No study.	[170]
*Chenomeles sinensis*	Polyphenols	Influenza virus	Inhibits the attachment of its hemagglutinins.	[172]
*Sambucus nigra*	Aqueous extract	Influenza virus	Modulates cytokine release and inhibits viral entrance.	[173]
*Phyllanthus emblica*	Aqueous extract	Influenza virus	Prevents hemagglutinins and viruses from infecting infected cells.	[174]
*Echinacea purpurea*	Aqueous extract	Influenza A/B viruses H3N2, H1N1, H5N1, H7N7, and S-OIV	Induces IL-6 and IL-8 production.	[177]
*Euphorbiacea shrub*	Polyphenolic polymers	Influenza	No study.	[181]
*Ficus religiosa*	Aqueous extract	Papillomavirus	HPV-16 apoptosis is induced.	[184]
*Bryophyllum pinnatum*	Chloroplast extract	Papillomavirus	Suppresses HPV-18 transcription.	[184]
*Pinellia pedatisecta*	Soluble extract	Papillomavirus	Inhibits the HPV-E6 expression in multiple cell lines.	[184]
*Carioca papaya*	Aqueous and chloroplast extract	Chikungunya	Stops the dengue virus.	[186]
*Sambucus nigra*	Methanolic extract	Dengue serotype 2	Defends against infection.	[187]
*Vernonia amygdalina*	Ethyl acetate extract	Chikungunya	Minimizes the viral burden.	[188]
*Picrorhiza kurrooa* *Ocimum tenuiflorum* *Terminalia chebula*	Aqueous extracts	Chikungunya	Block helicases and proteases.	[189]
*Lycoris radiate*, *Artemisia annua*, *Pyrrosia lingua*, *Lindera aggregata*, and *Isatis indigotica*	Different extracts	SARS-CoV-1	Obstruct 3CL protease.	[190]
*Rheum officinale Bail*, *Polygonum multiforum Thunb*	Different extracts	SARS-CoV-1	Inhibit the interaction between ACE2 and spike proteins.	[191]
*Gentiana scabana*, *Dioscorea batatas*, *Casssia tora*, *Taxillus chinensis*, and *Cibotium barometz*	Different extracts	SARS-CoV-1	Prevent 3CL protease.	[192]
*Anthemis hyalina*, *Nigella sativa*, *and Citrus sinensis*	Ethanolic extracts	SARS-CoV-1	Increase IL-8 and modulate gene expression of TRPA, TRPM, and TRPV.	[193]

**Table 6:** Antiviral biological compounds.

Secondary Metabolite Class	Biocompound (Species)	Virus	Potential Antiviral Mechanism	Reference
Menthane monoterpenoids	Carvacrol (**12**) (*Lippia graveolens*)	Herpes viruses	No study.	[125]
Furocoumarin	Imperatorin (**41**) and phellopterin (**42**) (*Angelica archangelica*)	Herpes simplex virus type 1 Coxsackievirus B3	No study.	[126]
Chromone	Eugenin (**43**) (*Geum japonicum*, *Syzygium aromaticum)*	Herpes simplex virus	Prevents DNA polymerase.	[127]
Cinnamic acid derivative	Rosmarinic acid (**47**) *(M. officinalis)*	Herpes simplex type 2	Prevents virus entry into cells.	[129]
Flavan-3-ol	Epigallocatechin-3-gallate (**48**) (*Camellia sinensis)*	Epstein–Barr Virus	Blocks transcription and protein expression via ERK1/2 (extracellular-regulated-kinase 12) and PI3-K/Akt (phosphatidylinositol-3-kinase) pathways.	[131]
Phenol, Monomeric stilbene	Sesamol (**49**), resveratrol (**2**) (*Sesamum indicum*)	Epstein–Barr Virus	Inhibit early antigen activation.	[132]
Isoquinoline alkaloid	Berberine (**15**) (*Barnerini vulgaris)*	Epstein–Barr Virus	Inhibits cell proliferation and induces apoptosis in Epstein–Barr virus-infected cells by inhibiting p-STAT3.	[134]
Linear diarylheptanoid	Curcumin (**33**) (*Curcuma longa)*	Epstein–Barr Virus	Inhibits TPA-, butyrate-, and TGF-b induced levels of BZLF1 mRNA	[135]
Flavone	Apigenin (**29**) (purchased from Sigma-Aldrich Co., St. Louis, MO, USA)	Epstein–Barr Virus	Inhibits lytic proteins Zta, Rta, EAD, and DNase in B and epithelial cells and reduces the production of EBV viruses.	[136]
Oleanane triterpenoid	Glycyrrhizic acid (**56**) (*Glycyrrhiza radix)*	Epstein–Barr Virus	Interferes with the initial phase of EBV replication.	[137]
Flavone	Luteolin (**25**) (purchased from Sigma-Aldrich Co.)	Epstein–Barr Virus	Inhibits the expression of proteins encoded by the EBV lytic gene.	[138]
Isoflavone	Genistein (**55**) (purchased from Sigma-Aldrich)	Cytomegalovirus	Inhibits immediate-early (ie) protein function.	[139]
Flavone	Baicalein (**57**) (purchased from Sigma-Aldrich)	Cytomegalovirus	Inhibits EGFR’s kinase activity to prevent viral entry.	[139]
Monomeric stilbene	Piceatannol (**58**) (purchased from Sigma-Aldrich)	Cytomegalovirus	Inhibits the lytic modifications and expression of hCMV early (E) and immediate–-early (IE) proteins.	[140]
Monomeric stilbene	Resveratrol (**2**) (purchased from Sigma-Aldrich)	Cytomegalovirus	Reduces DNA replication.	[141]
Sulfide	Allitridin (**59**) (*A. sativum)*	Cytomegalovirus	Inhibits the IE genes’ transcription.	[142]
Neolignan	Monoterpenylmagnolol (**52**) and β-eudesmol (**50**) (*Magnolia officinalis*)	Epstein–Barr Virus	Impede replication.	[133]
Cinnamic acid derivative	Isochlorogenic acid A (**61**) (*Laggera alata)*	Hepatitis virus B	Impedes replication.	[153]
Alkaloid	Amide alkaloids (*Piper longum*)	Hepatitis virus B	Inhibit replication and surface antigen expression.	[154]
Saponin	Saikosaponins (*Bupleurum* species)	Hepatitis virus B	Inhibit replication and surface antigen expression.	[156]
Protoberberine alkaloid	Dehydrocheilanthifoline (**60**) (*Corydalis saxifolia)*	Hepatitis virus B	Prevents reproduction.	[155]
Linear diarylheptanoid	Curcumin (**33**) (*Curcuma longa)*	Hepatitis virus B	Decreases Transcription.	[158]
Oleanane triterpenoid	Glycyrrhizinic acid (**56**) (*Glycyrrhiza glabra)*	Hepatitis virus B	Prevents viral reproduction.	[159,197]
Sesquiterpene lactone	Artemisinin (**62**) (*Artemisia annua)*	Hepatitis virus B	Prevents viral reproduction.	[159,197]
Isoflavonoid	LPRP-Et-97543 (**93**) (*Liriope platyphylla)*	Hepatitis virus B	Prevents viral reproduction.	[159,197]
Flavan-3-ol	Epigallocatechin-3-gallate (**48**) (*Camellia sinensis)*	Hepatitis virus B	Prevents viral reproduction.	[160]
Lignan	Flavonolignans (*Silybum marianum)*	Hepatitis C virus	No study.	[161]
Linear diarylheptanoid	Curcumin (**33**) (*Curcuma longa)*	Hepatitis C virus	Inhibits viral replication by blocking Akt-SREBP-1.	[162]
Flavan-3-ol	Epigallocatechin-3-gallate (**48**) (*Camellia sinensis*)	Hepatitis C virus	Inhibits viral introduction.	[163]
Flavone	Ladanein (**77**) (*Marrubium peregrinum*)	Hepatitis C virus	Inhibits viral introduction.	[164]
Peptide	Recombinant Griffithsin (*Nicotiana benthamiana*)	Hepatitis C virus	Inhibits viral cell–cell transmission.	[165]
Gallotannin	Tellimagrandin I (**78**) (*Rosae rugosae)*	Hepatitis C virus	Prevents viral penetration.	[159]
Benzopyran tannin and phenol	Chebulagic acid (**64**) and punicalagin (**65**) (*Terminalia chebula* Retz)	Hepatitis C virus	Inhibit fusion and cell–cell transmission.	[166]
Oleanane triterpenoid	Saikosaponin B2 (**79**) (*Bupleurum kaoi)*	Hepatitis C virus	Prevents viral attachment.	[159]
Furocoumarin, Quinoline alkaloid	Chalepine (**66**), pseudan IX (**80**) (*Ruta angustifolia)*	Hepatitis C virus	Reduce viral protein synthesis and viral RNA replication.	[159]
Lupane triterpenoids	Betulinic acid (**67**) and betulin (**68**) (*Betula alba* L)	Hepatitis C virus	Induce expression of TNF-α.	[168]
Oleanane triterpenoid	Glycyrrhizin (**56**) (*Glycyrrhiza glabra)*	Influenza virus	Initiates cell death in H5N1-infected cells.	[171]
Catechin	Catechins (*Camellia sinensis)*	Influenza virus	Inhibit both RNA synthesis and neuraminidase activity.	[175]
Dibenzylbutyrolactone lignans	Arctigenin (**69**) and arcitiin (**70**) (*Arctium lappa)*	Influenza virus	Anti-influenza A virus in vitro activity.	[176]
Monoterpenaldehydes	Citral a (**45**) and citral b (**46**) (*Melissa officinalis)*	H9N2 influenza virus	Have synergistic activity with oseltamivir.	[180]
Flavan-3-ols	Polyphenon E (poly E) (**71**) and epigallocatechin gallate (**48**) (*Camellia sinensis)*	Papillomavirus	Impede growth.	[182]
Sesquiterpene lactone	Artemisinin (**62**) (*Artemisia absintium)*	Papillomavirus	In ME-180 cells, this compound inhibits the expression of HPV-39, induces apoptosis, and reduces the proliferation of infected cells.	[183]
Tannin	Tannins (*Hamamelis virginiana)*	Papillomavirus	Inhibit HPV-16	[184]
Benzopyrone	Coumarin (**33**) (*Alternanthera philoxeroides)*	Chikungunya	Stops the dengue virus.	[185]
Anthraquinone, Stigmastane steroid, Flavanone, Anthranilic acid alkaloid, Glucosinolate	Emodin (**82**), β-sistosterol (**63**), hesperetin (**74**), indigo (**75**), and sinigrin (**76**) (*Isatis indigotica)*	SARS-CoV-1	Block the 3CL protease.	[194]
Flavones, Flavonol	Amentoflavone (**53**), apigenin (**29**), luteolin (**25**), quercetin (**10**) (*Torreya nucifera)*	SARS-CoV-1	Block the 3CL protease.	[195]
Indolizidine alkaloid	Lycorine (**54**) (*Lycoris radiata)*	SARS-CoV-1	Block 3CL protease.	[190]
Cinammic acid derivative, Anthraquinone, Oleanane triterpenoid, Flavonoid, Gallotannin	Caffeic acid (**51**), emodin (**82**), glycyrrhizin (**56**), luteolin (**25**), and tetra-O-galloyl-β-D-glucose (**81**)	SARS-CoV-2	Inhibit the spike protein’s interaction with the ACE-2 receptor.	[196]

#### 2.6.12. Molecules with Antiviral Activity Identified In Silico

Computational models enable us to simulate the interaction between the biocompound and the virus’s target molecule [198]. Quercetin-7-O-glucoside inhibits influenza virus RNA polymerase. Quercetagetin, a flavonoid with activity against HVC through the inhibition of RNA bound to NS5B non-structural polymerase [199], naringenin, and quercetin, could inhibit hepatitis C virus proteases [200], and β-amyrin could inhibit hepatitis D virus proteases [201].

Luteolin could block SARS-CoV-2 entrance into cells [202]; isothymol and curcumin can block angiotensin-converting enzyme receptor (ACE2) activity [203]; gingerol binds to the spike protein; and quercetin with proteases [204], enterodiol, taxifolin, eriodictyol, leucopelargonidin, morin, and myricetin were found to exhibit remarkable binding affinities against the major protease (Mpro) and potato-like protease (PLpro) [205].

## 3. Conclusions

The Latin American plant species studied in the last 20 years have shown various secondary metabolites and families of natural products that could be used to fight against antimicrobial resistance. Of particular interest, due to the events experienced by humanity in recent years, are antivirals. Many studies still need to be carried out to determine the structure–activity relationship of different compounds. However, it is assumed that natural products belonging to the same family will act similarly, but this still needs to be corroborated. The great wealth that Latin America presents regarding plant species variety can be used to benefit global health.

## Figures and Tables

**Table 3:** Mechanisms of action of families of antifungal drugs.

Family of Antifungal Drugs	Mechanism of Action	References
Azoles (fluconazole)	Inhibit fungal cell cytochrome P-450-3-A, disrupting ergosterol synthesis and intoxicating the cell with sterol intermediates.	[72,73,74]
Polyene (anfotericine B)	Binds to ergosterol and generates pores in the membrane, causing oxidative damage and cell death.	[72,73]
Echinocandins (micafungin)	Inhibit the enzyme 1,3-β-D-glucan synthase, which weakens the cell wall, causing osmotic instability.	[72,73]
Allylamines (terbinafine)	Block the enzyme squalene epoxidase, reducing ergosterol levels and increasing squalene. This increases the permeability of the cell. membrane, causing a decrease in fungal growth.	[72]
Pyrimidines (flucytosine)	Bind to cytosine permease, already in the nucleus, and generate fluorardilic acid, which is incorporated into the RNA, rendering it useless.	[72,74]
Orotomides (olorofim)	Inhibit dihydroorotate dehydrogenase synthesis, preventing the synthesis of DNA and RNA.	[76,77]
Fosmanogepix	Inhibits the enzyme Gwt1, responsible for glycosylphosphatidylinositol synthesis.	[75]

**Table 4:** Extracts of Latin American plants with activity against pathogenic fungi.

Species	Extract	Fungi	References
*Achyrocline satureioides*	Ethanolic	*Fusarium verticillioides*	[82]
*Achyrocline tomentosa*	Ethanolic	*Fusarium verticillioides*	[82]
*Aloysia citriodora*	Ethanolic	*Fusarium verticillioides*	[82]
*Annona cherimola*	Ethanolic	*Fusarium oxysporum*	[83]
*Annona muricata* L.	Ethanolic	*Candida albicans*	[84]
*Aristolochia argentina Griseb.*	Ethanolic	*Fusarium verticillioides*	[82,85]
*Asclepias curassavica*	Hexanic, Methanolic	*Candida albicans*	[86]
*Baccharis artemisioides*	Ethanolic	*Fusarium verticillioides*	[82]
*Baccharis flabellata*	Ethanolic	*Fusarium verticillioides*	[82]
*Baccharis salicifolia*	Ethanolic	*Fusarium verticillioides*	[82]
*Bixa orellana*	Ethanolic	*Candida albicans*	[87]
*Curcuma zedoaria*	Acetone, Hexanic	*Candida albicans*	[88,89]
*Dalea elegans*	Ethanolic	*Fusarium verticillioides*	[82]
*Echinacea angustifolia*	Ethanolic	*Cryptococcus neoformans*	[90]
*Echinacea atrorubens*	Ethanolic	*Cryptococcus neoformans*	[91]
*Echinacea pallida*	Ethanolic	*Candida albicans*	[91]
*Echinacea purpurea*	Ethanolic	*Saccharomyces cerevisiae*	[90]
*Eupatorium buniifolium*	Methanolic	*Trichophyton mentagrophytes*	[92]
*Euphorbia hyssopifolia*	Methanolic	*Aspergillus niger*	[93]
*Flourensia oolepis*	Ethanolic	*Fusarium verticillioides*	[82]
*Gaillardia megapotamica*	Ethanolic	*Fusarium verticillioides*	[82]
*Galphimia glauca*	Hexanic, Methanolic	*Trichophyton mentagrophytes*	[86,94]
*Grindelia pulchella*	Ethanolic	*Fusarium verticillioides*	[82]
*Heterothalamus alienus*	Ethanolic	*Fusarium verticillioides*	[82]
*Hibiscus sabdariffa*	Methanolic	*Candida albicans*	[95]
*Kageneckia lanceolata*	Ethanolic	*Fusarium verticillioides*	[82]
*Larrea cuneifolia*	Ethanolic	*Lenzites elegans*	[96]
*Larrea divaricata*	Ethanolic	*Penicillium notatum*; *Candida* spp.	[96,97]
*Lepechinia floribunda*	Ethanolic	*Fusarium verticillioides*	[82]
*Lippia turbinata*	Ethanolic	*Fusarium verticillioides*	[82]
*Loeselia mexicana*	Ethanolic	*Trichophyton mentagrophytes*	[98]
*Lygodium venustum*	Ethanolic	*Candida albicans*	[99]
*Lysiloma acapulcensis*	Hexanic	*Trichophyton mentagrophytes*	[100]
*Miconia mexicana*	Methanolic	*Candida albicans*	[100]
*Microliabum candidum*	Ethanolic	*Fusarium verticillioides*	[82]
*Minthostachys verticillata*	Ethanolic	*Fusarium verticillioides*	[82]
*Morrenia brachystephana*	Ethanolic	*Fusarium verticillioides*	[82]
*Otholobium higuerilla*	Ethanolic	*Fusarium verticillioides*	[82]
*Passiflora caerulea*	Methanolic	*Aspergillus flavus*	[101]
*Pimenta dioica*	Essential oil	*Fusarium oxysporum*	[102]
*Polygonum acuminatum*	Dichloromethane	*Cryptococcus neoformans*	[103]
*Salix alba*	Methanolic	*Aspergillus ornatus*	[104]
*Salvia cuspidata*	Ethanolic	*Fusarium verticillioides*	[82]
*Sebastiania commersoniana*	Ethanolic	*Candida* spp.	[105]
*Senecio vira-vira*	Ethanolic	*Fusarium verticillioides*	[82]
*Smilax domingensis*	Ethanolic	*Candida albicans*	[106]
*Syzygium aromaticum*	Essential oil	*Candida* spp.	[107]
*Terminalia triflora*	Methanolic	*Trichophyton mentagrophytes*	[92]
*Thalictrum decipiens*	Ethanolic	*Fusarium verticillioides*	[82]
*Tithonia diversifolia*	Aquous	*Fusarium oxysporum*	[108]
*Trichocline reptans*	Ethanolic	*Fusarium verticillioides*	[82]
*Vernonia mollisima*	Ethanolic	*Fusarium verticillioides*	[82]
*Vernonia nudiflora*	Ethanolic	*Fusarium verticillioides*	[82]
*Vitis vinifera*	Aqueous	*Candida* spp.	[109]
*Zanthoxylum coco*	Ethanolic	*Fusarium verticillioides*	[82]
*Zinnia peruviana*	Ethanolic	*Fusarium oxysporum*	[96]
*Zuccagnia punctata*	Ethanolic	*Aspergillus niger*	[96]
*Zuccagnia punctata*	Dichloromethane	*Candida albicans*	[110]
